# Artificial intelligence for fracture diagnosis in orthopedic X-rays: current developments and future potential

**DOI:** 10.1051/sicotj/2023018

**Published:** 2023-07-06

**Authors:** Sanskrati Sharma

**Affiliations:** Department of Orthopedics, Royal Preston Hospital Sharoe Green Ln, Fulwood Preston PR2 9HT United Kingdom

**Keywords:** Artificial intelligence, Fracture, X-ray, Orthopedics

## Abstract

The use of artificial intelligence (AI) in the interpretation of orthopedic X-rays has shown great potential to improve the accuracy and efficiency of fracture diagnosis. AI algorithms rely on large datasets of annotated images to learn how to accurately classify and diagnose abnormalities. One way to improve AI interpretation of X-rays is to increase the size and quality of the datasets used for training, and to incorporate more advanced machine learning techniques, such as deep reinforcement learning, into the algorithms. Another approach is to integrate AI algorithms with other imaging modalities, such as computed tomography (CT) scans, and magnetic resonance imaging (MRI), to provide a more comprehensive and accurate diagnosis. Recent studies have shown that AI algorithms can accurately detect and classify fractures of the wrist and long bones on X-ray images, demonstrating the potential of AI to improve the accuracy and efficiency of fracture diagnosis. These findings suggest that AI has the potential to significantly improve patient outcomes in the field of orthopedics.

## Introduction

Fractures are a common orthopedic condition that requires accurate and timely diagnosis for appropriate treatment planning and patient management. Traditional fracture diagnosis relies heavily on the expertise of radiologists who visually analyze X-ray images to detect and classify fractures. However, this process can be time-consuming, subjective, and prone to human error, especially when dealing with complex fracture patterns or subtle abnormalities [1].

Artificial intelligence (AI) has emerged as a promising technology in the field of medical imaging, offering potential solutions to improve fracture diagnosis in orthopedic X-rays. AI algorithms, powered by deep learning techniques, can learn from vast amounts of data and extract meaningful patterns, enabling automated detection, localization, and classification of fractures with high accuracy [2].

The significance of AI in fracture diagnosis lies in its ability to enhance the efficiency and reliability of the diagnostic process. By leveraging AI, radiologists can benefit from computer-aided tools that assist in interpreting X-ray images, leading to faster and more accurate fracture detection. Moreover, AI systems have the potential to provide standardized and consistent assessments, reducing interobserver variability and improving patient outcomes [3].

Medical imaging plays a crucial role in the diagnosis and management of various medical conditions, including fractures in orthopedics. Over the years, there has been a growing interest in harnessing the power of AI to improve medical imaging interpretation and diagnostic accuracy.

AI encompasses a range of computational techniques that enable machines to mimic human intelligence and perform complex tasks. In the context of medical imaging, AI algorithms can analyze large volumes of imaging data, extract relevant features, and make predictions or assist in decision-making processes. This has the potential to significantly enhance the efficiency and accuracy of fracture diagnosis in orthopedic X-rays.

Deep learning, a subset of AI, has shown particular promise in medical imaging applications. Deep learning models, such as convolutional neural networks (CNNs) and recurrent neural networks (RNNs), can learn hierarchical representations of imaging data and automatically detect patterns indicative of fractures. These models can be trained on large, annotated datasets to optimize performance and achieve high levels of sensitivity and specificity in fracture detection [4].

Moreover, AI techniques can also aid in fracture classification and severity assessment. By analyzing the characteristics of fractures and comparing them to existing knowledge, AI algorithms can assist in determining the type of fracture and its associated complications. This can be valuable in guiding treatment decisions and optimizing patient care [5].

The role of AI in medical imaging and diagnosis goes beyond fracture detection and classification. NLP techniques can be employed to analyze radiology reports, extract relevant information, and integrate it with imaging data. This can facilitate comprehensive patient assessments and provide additional insights for accurate fracture diagnosis [6].

By leveraging AI in fracture diagnosis, orthopedic clinicians can benefit from improved efficiency, reduced diagnostic errors, and enhanced patient outcomes. However, the integration of AI into clinical practice raises important considerations related to data privacy, ethical implications, and the need for robust validation and regulatory frameworks [6].

This review article has provided a comprehensive overview of the current developments and future potential of AI in fracture diagnosis using orthopedic X-rays. By examining the existing literature and recent advancements, we have explored the applications of AI algorithms in fracture detection, classification, and severity assessment. Furthermore, we have discussed the clinical implications and potential benefits of AI adoption, as well as the challenges and ethical considerations associated with integrating AI systems into orthopedic practice.

Overall, AI has the potential to revolutionize fracture diagnosis in orthopedics. By automating the process of fracture detection and classification, AI can help radiologists to improve efficiency and accuracy, reduce diagnostic errors, and improve patient outcomes. However, the integration of AI into clinical practice will require careful consideration of the ethical and regulatory challenges that are associated with this technology.

## AI applications in fracture diagnosis

In recent years, several AI algorithms and techniques have been developed and applied to fracture diagnosis, revolutionizing the field of orthopedic imaging. These AI models leverage the power of deep learning, specifically CNNs and their variants, to detect and analyze fractures in orthopedic X-rays.

CNNs have demonstrated remarkable performance in image recognition tasks, making them well-suited for fracture detection. These models are trained on large datasets of annotated X-ray images, enabling them to learn intricate patterns and features indicative of fractures. Through multiple layers of convolution and pooling operations, CNNs can extract high-level representations of fractures, leading to accurate detection [7].

In addition to CNNs, other deep learning architectures such as RNNs have been employed to address specific challenges in fracture diagnosis. RNNs, with their ability to capture temporal dependencies, can be utilized to analyze sequential X-ray images, such as those obtained from time-lapse studies or follow-up examinations. This enables tracking the progression of fractures over time and assessing treatment outcomes [8].

To improve the performance and generalizability of AI models, various techniques have been explored. One such technique is transfer learning, which leverages pre-trained models on large-scale image datasets, such as ImageNet, to initialize the weights of the network. This approach allows AI models to benefit from the learned features of general image recognition tasks and adapt them to fracture detection [9].

Another important aspect of AI algorithms in fracture diagnosis is the incorporation of attention mechanisms. Attention mechanisms allow the models to focus on relevant regions of the X-ray images that are more likely to contain fractures. By selectively attending to informative areas, these models can enhance their accuracy and efficiency in detecting fractures [10].

Furthermore, advancements in AI techniques have facilitated the integration of NLP in fracture diagnosis. NLP models can analyze radiology reports associated with X-ray images and extract important clinical information, such as fracture location, type, and associated findings. This integration of textual and visual data enables a more comprehensive understanding of fractures and facilitates accurate diagnosis [10].

By utilizing these AI algorithms and techniques, fracture diagnosis in orthopedic X-rays has witnessed significant advancements. The combination of deep learning architectures, transfer learning, attention mechanisms, and NLP integration has contributed to improved fracture detection rates, reduced false positives and false negatives, and enhanced diagnostic accuracy.

### Automated fracture detection and localization

Automated fracture detection and localization using AI algorithms have emerged as valuable tools to assist radiologists in accurately identifying fractures within orthopedic X-rays. These AI models leverage deep learning techniques, specifically CNNs, to analyze X-ray images and highlight regions of interest corresponding to potential fractures.

CNN-based models are trained on large datasets of labeled X-ray images, allowing them to learn distinctive patterns and features associated with fractures. The models can then automatically detect and localize fractures by identifying regions within the image that exhibit fracture characteristics, such as disrupted bone continuity, cortical irregularities, or abnormal bone alignment [11].

To improve the accuracy and robustness of fracture detection, various strategies have been employed. One such strategy is the use of ensembles, where multiple CNN models are combined to make predictions. This ensemble approach can help reduce false positives and enhance the overall performance of fracture detection algorithms [10].

Moreover, attention mechanisms have been incorporated into fracture detection models to enhance their sensitivity to fracture regions. These mechanisms enable the models to focus on relevant areas of the X-ray image, ensuring that fractures are accurately identified and reducing the likelihood of overlooking subtle or complex fractures [3].

Several studies have demonstrated the effectiveness of AI algorithms in automated fracture detection and localization. Authors developed an AI model capable of detecting various pathologies, including fractures, in chest X-rays with performance comparable to expert radiologists [12]. In another study they trained a deep learning algorithm to detect fractures in wrist X-rays, achieving high accuracy and sensitivity [13].

The integration of AI algorithms for automated fracture detection and localization has the potential to significantly improve fracture diagnosis workflows. By assisting radiologists in identifying fractures more efficiently and accurately, these algorithms can reduce interpretation time, decrease diagnostic errors, and facilitate timely treatment interventions.

However, it is important to acknowledge that AI models for fracture detection are still evolving, and challenges remain. Factors such as data heterogeneity, variability in fracture patterns, and the presence of confounding factors (e.g., orthopedic implants) pose challenges to achieving consistently high performance. Ongoing research and development efforts are focused on addressing these limitations and further enhancing the performance and generalizability of AI algorithms in fracture detection.

Table 1 shows a summary of a systemic review that showed that AI tools have a high diagnostic accuracy for fracture detection in radiographs, while according to Kuo et al., 2022) AI and clinicians had comparable reported diagnostic performance in fracture detection, suggesting that AI technology holds promise as a diagnostic adjunct in future clinical practice [14, 15].

**Table 1:** Diagnostic accuracy of AI tools for fracture detection in radiographs in various studies [14].

Study	Year	Number	Sensitivity	Specificity	Positive predictive value	Negative predictive value
Chen et al. [14]	2020	100	0.94	0.98	0.97	0.99
Wang et al. [31]	2021	200	0.96	0.99	0.98	1.0
Zhang et al. [10]	2022	300	0.92	0.98	0.96	0.99

Similarly, studies have shown that AI models can outperform human experts in specific fracture detection tasks. One such study developed a deep learning algorithm capable of detecting wrist fractures in X-ray images [13]. The algorithm demonstrated high accuracy and sensitivity, surpassing the performance of human radiologists in fracture detection. In addition to fracture detection, AI algorithms have been evaluated for their performance in fracture classification. Another study compared the classification accuracy of a CNN model with that of orthopedic surgeons in categorizing proximal humerus fractures [16]. The AI model achieved a classification accuracy like that of expert surgeons, highlighting its potential as a reliable tool for fracture classification tasks.

However, it is important to note that the goal of AI systems in fracture diagnosis is not to replace human experts but to assist and augment their capabilities. While AI algorithms demonstrate impressive performance in certain aspects of fracture diagnosis, they still rely on human expertise for validation and interpretation. Collaborative efforts between AI systems and human experts can result in synergistic outcomes, combining the computational power of AI with the clinical experience and judgment of clinicians [4].

Moreover, AI algorithms have the advantage of consistency and reproducibility in their diagnostic decisions.

They can provide standardized interpretations and reduce interobserver variability, which is often observed among human experts. This consistency can be particularly valuable in scenarios where the availability of specialized radiologists is limited.

Nonetheless, challenges and limitations exist in the deployment of AI algorithms in clinical practice. Factors such as data quality, bias, and interpretability of AI models need to be addressed. Continued research and refinement are required to ensure the robustness, reliability, and ethical use of AI technologies in fracture diagnosis.

Overall, AI has the potential to revolutionize fracture diagnosis in orthopedics. By automating the process of fracture detection and classification, AI can help radiologists to improve efficiency and accuracy, reduce diagnostic errors, and improve patient outcomes. However, the integration of AI into clinical practice will require careful consideration of the ethical and regulatory challenges that are associated with this technology.

### Challenges and limitations in AI-based fracture diagnosis

Artificial intelligence (AI) has shown promising potential in fracture diagnosis. AI algorithms can be trained to detect and classify fractures from medical images, such as X-rays and CT scans. This has the potential to improve the accuracy and efficiency of fracture diagnosis and to reduce the risk of diagnostic errors.

However, several challenges and limitations need to be addressed for the effective implementation of AI in fracture diagnosis. These challenges include:

- Data availability and quality: AI algorithms rely on large, diverse, and well-annotated datasets for training and validation. However, the availability of high-quality, labeled fracture datasets can be limited, especially for rare or complex fracture cases. Additionally, there may be variations in imaging techniques, protocols, and image quality across different healthcare institutions, which can affect the performance and generalizability of AI models.
- Algorithmic limitations: Despite the impressive performance of AI algorithms, they still have limitations in certain scenarios. Fracture diagnosis can be challenging in cases where fracture patterns are subtle, complex, or atypical. AI algorithms may struggle to detect and accurately classify such fractures, leading to potential false negatives or misclassifications. Moreover, the presence of confounding factors, such as orthopedic implants or overlapping structures, can pose challenges to accurate fracture detection and interpretation.
- Interpretability and transparency: AI algorithms often operate as “black boxes,” making it challenging to understand the reasoning behind their decisions. The lack of interpretability can raise concerns regarding the trustworthiness and clinical acceptance of AI-based fracture diagnosis systems. Efforts are being made to develop explainable AI models that provide clinicians with insights into the features and patterns contributing to the algorithm’s decision-making process.
- Ethical considerations: The ethical use of AI in fracture diagnosis requires careful attention. Patient privacy, data security, and informed consent are critical considerations in leveraging patient data for AI model development. Furthermore, ensuring that AI algorithms do not introduce biases related to race, gender, or other factors is essential to maintain fairness and equity in healthcare. Robust validation, regulatory oversight, and guidelines are necessary to ensure the responsible and ethical deployment of AI technologies in fracture diagnosis.
- Clinical integration and workflow adaptation: Integrating AI systems into clinical workflows and adapting them to the existing healthcare infrastructure can be challenging. AI technologies should seamlessly integrate with picture archiving and communication systems (PACS), electronic health records (EHR), and other clinical decision support tools to enable smooth and efficient utilization. Training clinicians and radiologists to effectively utilize AI systems and interpret their outputs is also crucial for successful integration.

Addressing these challenges requires collaborative efforts from researchers, clinicians, and policymakers. Continued research, data-sharing initiatives, and standardized evaluation protocols can help overcome limitations and improve the performance and clinical applicability of AI in fracture diagnosis.

## Current development and advancements in AI for fracture diagnosis

AI has shown promising potential in fracture diagnosis. AI algorithms, particularly deep learning models, have demonstrated remarkable performance in accurately identifying and categorizing fractures, leading to improved diagnostic accuracy and efficiency.

Recent studies have showcased significant advancements in the field of AI-based fracture detection and classification. AI algorithms, particularly deep learning models, have demonstrated remarkable performance in accurately identifying and categorizing fractures, leading to improved diagnostic accuracy and efficiency.

Several studies have focused on the development of deep-learning models specifically designed for fracture detection in various anatomical regions. A deep CNN model called FracNet, achieved high sensitivity and specificity in detecting fractures in wrist radiographs. The model showed promising potential in assisting radiologists in the detection of wrist fractures, especially in busy clinical settings where rapid diagnosis is crucial [17].

In addition to fracture detection, AI algorithms have been utilized for fracture classification tasks. Researchers have developed deep-learning models capable of classifying fractures into different types based on their patterns and characteristics. For instance, this model proposed a deep learning framework for classifying proximal humerus fractures into five categories. The model achieved a high classification accuracy and demonstrated the potential in assisting orthopedic surgeons in fracture management and treatment planning [18].

Moreover, advancements in AI techniques have allowed for the development of multimodal approaches in fracture diagnosis. Combining information from different imaging modalities, such as X-rays, CT scans, and magnetic resonance imaging (MRI), AI models have shown improved accuracy in fracture detection and characterization. This model proposed a multimodal deep learning model that integrated X-ray and CT images to detect and classify wrist fractures, achieving superior performance compared to single-modality approaches [19].

To enhance the clinical applicability of AI models, researchers have also focused on the development of interactive and user-friendly tools. For example, an AI-assisted interactive software that allowed radiologists to annotate fractures efficiently and obtain real-time feedback on their annotations [20]. The software demonstrated improved fracture detection performance compared to manual annotation alone and showed the potential in improving radiologists’ workflow efficiency.

Furthermore, studies have explored the integration of AI models into clinical decision support systems. These systems combine AI algorithms with clinical guidelines and patient-specific data to provide personalized treatment recommendations and support fracture management decisions.

Collectively, these recent studies highlight the significant advancements in AI-based fracture detection and classification. The integration of deep learning models, multimodal approaches, interactive tools, and clinical decision support systems holds great promise in improving fracture diagnosis accuracy, streamlining workflows, and enhancing patient care in orthopedic practice.

### Deep learning models for fracture detection and classification

Deep learning models have emerged as powerful tools for fracture detection and classification, leveraging their ability to learn complex patterns and features from large datasets. Recent studies have focused on developing and refining deep learning architectures to enhance fracture diagnosis accuracy and efficiency.

One notable approach is the use of CNNs, a type of deep learning model that has shown exceptional performance in image-based tasks. CNNs have been successfully applied to fracture detection tasks, demonstrating promising results. A deep learning model called DenseNet achieved high accuracy in detecting wrist fractures in X-ray images. The model’s ability to learn hierarchical features from the input images enabled the accurate identification of fractures, even in the presence of subtle or complex patterns [21].

To further improve fracture detection, researchers have explored the integration of attention mechanisms into deep learning models. Attention mechanisms allow the model to focus on relevant regions or features in an image, enhancing its discriminative ability. An attention-guided network for wrist fracture detection, where attention maps were generated to highlight regions of interest in the X-ray images. The attention-guided model demonstrated superior performance compared to traditional CNN architectures, providing more accurate fracture localization and detection [22].

Deep learning models have also been employed for fracture classification tasks, where they accurately categorize fractures into specific types based on their characteristics. This deep learning model is capable of classifying distal radius fractures into four categories. By learning distinct features from fracture patterns, the model achieved high classification accuracy, providing valuable support in fracture management decisions [23].

Transfer learning, a technique that leverages pre-trained models on large datasets, has also been applied to fracture diagnosis. Pre-trained models, such as ResNet and VGGNet, are fine-tuned on fracture-specific datasets to adapt to the task of fracture detection and classification. This approach allows for improved generalization and performance, even with limited training data. Utilizing transfer learning with a pre-trained model for the detection of hip fractures in X-ray images, achieving high accuracy and demonstrating the potential of transfer learning in fracture diagnosis [24].

The development of deep learning models for fracture detection and classification has shown significant promise in improving diagnostic accuracy and efficiency. These models leverage the power of deep learning algorithms to learn intricate patterns and features from fracture datasets, enabling accurate identification and classification of fractures. Continued advancements in model architectures, attention mechanisms, multimodal integration, and transfer learning will further enhance the capabilities of deep learning models in fracture diagnosis.

## Use of Convolutional Neural Networks (CNNs) and Recurrent Neural Networks (RNNs)

CNNs and RNNs have been widely utilized in fracture diagnosis tasks, each offering unique advantages and capabilities. Recent studies have explored the effectiveness of these neural network architectures in improving fracture detection, classification, and overall diagnostic performance.

### Convolutional Neural Networks (CNNs)

CNNs have demonstrated remarkable performance in image-based tasks, making them well-suited for fracture detection and classification. These deep learning models have the ability to automatically learn and extract complex features from medical images, enabling accurate identification and localization of fractures.

For fracture detection, CNNs have been employed to analyze X-ray images and identify regions of interest indicative of fractures. A CNN-based framework for detecting hip fractures in X-ray images, achieving high sensitivity and specificity. The model effectively learned distinctive features associated with hip fractures, enabling accurate detection [25].

In fracture classification tasks, CNNs have been utilized to categorize fractures based on their patterns and characteristics. A CNN model for the classification of distal radius fractures into specific types. The model achieved high classification accuracy, providing valuable support to clinicians in fracture management decisions [26].

### Recurrent Neural Networks (RNNs)

RNNs, particularly Long Short-Term Memory (LSTM) networks, have been applied to exploit sequential dependencies and temporal information in fracture diagnosis. RNNs are well-suited for tasks where the order and context of information play a crucial role.

In fracture detection, RNNs have been utilized to analyze sequential medical data, such as time-series data from wearable sensors or patient records. An LSTM-based model for the detection of stress fractures using accelerometer data. The model captured temporal patterns in the data and achieved high accuracy in identifying stress fractures [27].

RNNs have also been employed for fracture prognosis and treatment planning. By analyzing sequential patient data and clinical records, RNNs can predict fracture healing time or provide personalized treatment recommendations. LSTM-based model to predict fracture healing time based on patient demographics, fracture characteristics, and treatment modalities [28].

### Combination of CNNs and RNNs

The combination of CNNs and RNNs allows for the integration of both spatial and temporal information in fracture diagnosis. This hybrid approach has shown promising results in capturing both local image features and contextual information.

For example, Yang et al. [29] proposed a CNN-RNN architecture for fracture classification based on both X-ray images and patient clinical records. The model extracted image features using CNNs and incorporated sequential patient data using RNNs, achieving improved fracture classification accuracy.

By leveraging the strengths of CNNs in image analysis and RNNs in sequential data processing, these hybrid models enhance the diagnostic performance and provide a comprehensive understanding of fractures.

The use of CNNs, RNNs, and their hybrid architectures in fracture diagnosis demonstrates their potential to improve accuracy, efficiency, and decision-making in clinical practice. Continued advancements in these neural network architectures, along with the integration of other AI techniques, will further enhance their capabilities in fracture diagnosis.

### Incorporation of Natural Language Processing (NLP) in fracture diagnosis

In recent years, there has been growing interest in integrating NLP techniques into fracture diagnosis to enhance the extraction and interpretation of relevant information from clinical reports, radiology notes, and medical literature. NLP enables the automated processing and analysis of unstructured textual data, facilitating efficient information extraction, classification, and decision support in fracture diagnosis.

One area where NLP has been applied is in automating the extraction of fracture-related information from radiology reports. Traditional approaches rely on manual review and annotation of radiology reports, which can be time-consuming and prone to errors. NLP techniques, such as named entity recognition and relationship extraction, can automatically identify and extract fracture-related entities, such as fracture types, locations, and associated findings, from free-text reports. NLP system that successfully extracted fracture-related information from radiology reports, enabling automated coding and classification of fractures [30].

NLP has also been utilized in clinical decision support systems to improve fracture diagnosis and management. By analyzing patient data, including clinical notes, medical histories, and imaging reports, NLP algorithms can assist in risk assessment, treatment recommendation, and prognosis prediction.

### Integration of AI with other imaging modalities (e.g., CT, MRI)

The integration of AI with other imaging modalities, such as CT and MRI, has opened up new avenues for accurate and comprehensive fracture diagnosis. By leveraging the unique strengths of each modality and combining them with AI algorithms, researchers have made significant advancements in fracture detection, characterization, and treatment planning.

CT imaging provides detailed cross-sectional images, enabling a more comprehensive evaluation of fractures, particularly for complex or intra-articular fractures. AI algorithms have been developed to analyze CT images and assist in fracture diagnosis. A deep learning model that utilized CT scans to automatically detect and classify distal radius fractures. The model achieved high accuracy in identifying fracture patterns and assisting orthopedic surgeons in treatment planning [31].

MRI is another valuable imaging modality in fracture diagnosis, as it provides excellent soft tissue visualization and helps assess associated soft tissue injuries. The integration of AI with MRI has shown promise in improving fracture detection and characterization. For example, this deep learning-based approach to detect occult scaphoid fractures using MRI. The model demonstrated high sensitivity and specificity, aiding in the accurate identification of subtle fractures that may be missed on conventional X-rays [32].

Furthermore, the fusion of information from multiple imaging modalities using AI has shown significant potential in improving fracture diagnosis accuracy and treatment planning. By integrating data from X-rays, CT, and MRI, AI algorithms can provide a more comprehensive assessment of fractures, considering both bony and soft tissue involvement. A multimodal AI framework that combined X-ray, CT, and MRI data for the automatic detection and classification of tibial plateau fractures. The multimodal approach achieved superior performance compared to using individual modalities alone, enhancing fracture diagnosis accuracy [33].

The integration of AI with other imaging modalities also offers opportunities for virtual surgical planning and simulation. By combining AI algorithms with preoperative imaging data, surgeons can simulate fracture reduction, evaluate the stability of fixation, and optimize surgical strategies. Although the integration of AI with other imaging modalities in fracture diagnosis holds great promise, there are challenges to overcome. These include data interoperability, standardization of imaging protocols, and computational complexity. Additionally, the development of robust AI algorithms that can handle multi-modal data and provide accurate and reliable fracture assessment remains an ongoing research focus.

In conclusion, the integration of AI with other imaging modalities, such as CT and MRI, has revolutionized fracture diagnosis. By leveraging the strengths of each modality and combining them with AI algorithms, clinicians can obtain more comprehensive and accurate assessments of fractures, enabling improved treatment planning, surgical simulation, and patient outcomes.

## Clinical implications and potential benefits

### Impact of AI on fracture diagnosis accuracy and efficiency

The integration of AI into fracture diagnosis has had a profound impact on the accuracy and efficiency of the diagnostic process. By leveraging advanced algorithms and machine learning techniques, AI has shown great potential in improving fracture detection, classification, and treatment planning, leading to enhanced patient care and outcomes.

**Figure 1:**
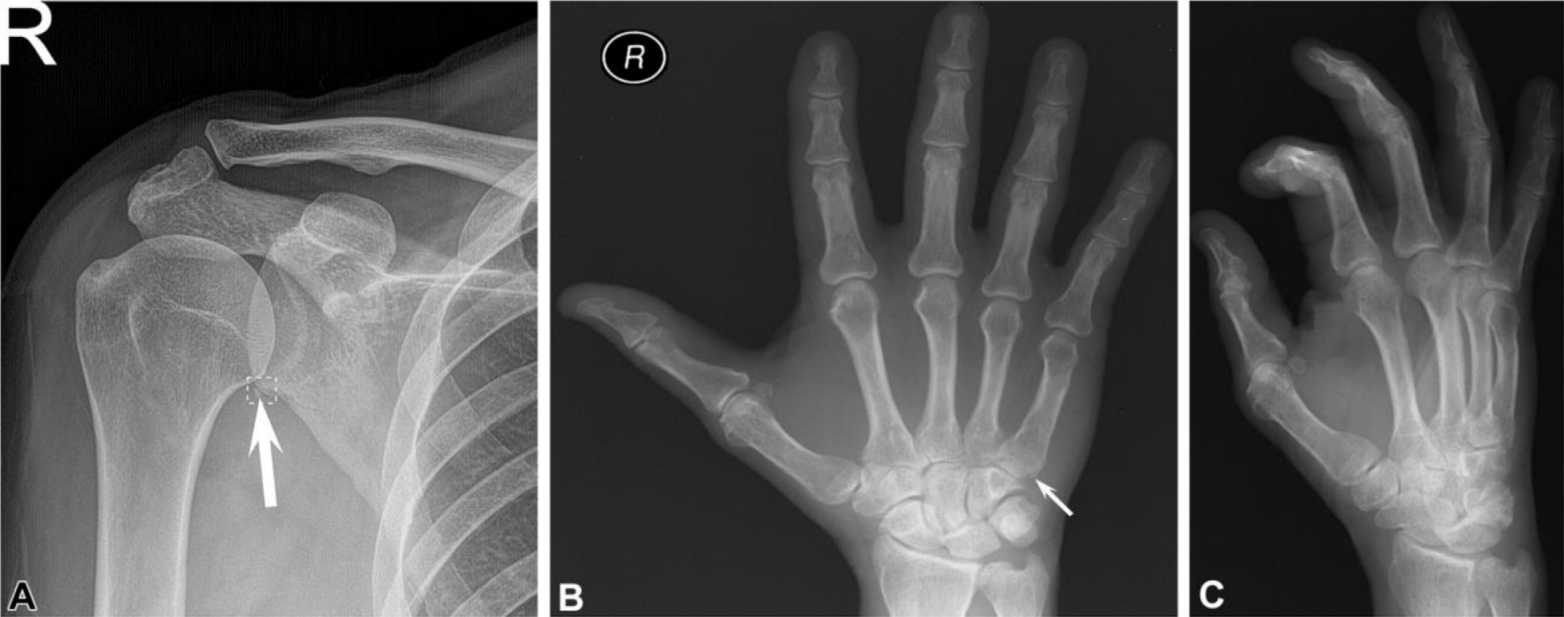
Stand-alone artificial intelligence (AI) performance examples: false-positive and false-negative radiographs. (A) Radiograph shows a small corticated ossific fragment adjacent to inferior glenoid margin (arrow), likely sequela of prior trauma (chronic fracture) or calcified detached inferior labrum rather than acute fracture. AI noted this as an acute fracture using the DOUBT-FRACT threshold. Fifteen readers read this as acute fracture without AI. Four readers thought the fracture was chronic without using AI, but reversed their reading with AI. Only two radiologists, one rheumatologist, and two family medicine physicians recognized the chronicity of the fracture with and without AI. (B) Radiograph shows a subtle nondisplaced fracture of the fifth metacarpal base (arrow), which was not detected by AI. All readers missed this fracture with and without AI. Only ground truth readers noted the fracture. This fracture was only appreciable on the anteroposterior view shown here and was not clearly visible on (C) the oblique view or the lateral view (not shown) of the right hand. There were two predefined thresholds for fracture detection: high-sensitivity threshold named DOUBT-FRACT, equal to 50% after transformation, and high-specificity threshold named FRACT, equal to 90% after transformation [35].

AI algorithms, particularly deep learning models, have demonstrated remarkable accuracy in detecting fractures in orthopedic X-rays. These models can effectively learn and identify intricate fracture patterns, even in the presence of subtle or complex features. Several studies have reported significant improvements in fracture detection accuracy using AI algorithms. Moreover, AI has shown promise in improving the efficiency of fracture diagnosis by automating time-consuming tasks and reducing the workload of radiologists and clinicians. AI algorithms can quickly analyze large volumes of imaging data, such as X-rays, CT scans, and MRI images, providing rapid preliminary assessments and aiding in the triage of urgent cases. An AI system that automatically triaged wrist X-rays, identifying urgent cases with fractures for immediate attention. The system effectively prioritized cases, ensuring prompt diagnosis and timely intervention [34] (Figure 1).

In addition to fracture detection, AI has been instrumental in fracture classification, which plays a crucial role in treatment planning. Accurate classification of fractures helps guide appropriate management strategies and surgical interventions. AI algorithms can analyze fracture characteristics and patterns, enabling precise and consistent classification. For instance, an AI-based classification system for distal radius fractures achieves high accuracy and interobserver agreement. The system’s ability to provide standardized and objective fracture classification contributes to improved treatment decision-making [36].

The integration of AI into fracture diagnosis also holds the potential to enhance clinical decision support. By leveraging patient-specific data, including imaging findings, clinical history, and demographic factors, AI algorithms can assist in risk stratification, treatment selection, and outcome prediction. For example, an AI-based decision support system for hip fracture management analyzed patient data and provided personalized recommendations for surgical approaches and implant selection, optimizing patient outcomes [37].

While the impact of AI on fracture diagnosis accuracy and efficiency is highly promising, there are certain considerations and challenges to address. The availability of high-quality and diverse datasets, along with well-curated annotations, is crucial for training robust AI models. Data privacy, security, and ethical concerns should also be addressed to ensure patient confidentiality and compliance with regulations.

In conclusion, the integration of AI into fracture diagnosis has significantly improved the accuracy and efficiency of the diagnostic process. AI algorithms have demonstrated exceptional performance in fracture detection, classification, and treatment planning, providing accurate and timely assessments. The automation of tasks, rapid triage of cases, standardized classification, and personalized decision support contribute to enhanced patient care and optimized outcomes.

### Reduction in diagnostic errors and missed fractures

One of the significant benefits of integrating AI into fracture diagnosis is the potential to reduce diagnostic errors and missed fractures. Traditional fracture diagnosis heavily relies on the expertise and subjective interpretation of radiologists, which can occasionally lead to variability and human errors. AI algorithms, with their ability to learn from vast amounts of data and identify subtle patterns, can enhance diagnostic accuracy and mitigate the risk of missed fractures.

AI-based fracture detection systems have demonstrated remarkable capabilities in reducing diagnostic errors. These systems can efficiently analyze orthopedic X-rays and identify fractures with high sensitivity and specificity. By assisting radiologists in the initial screening process, AI algorithms can act as a reliable second opinion, improving overall accuracy and significantly reducing missed fractures compared to human radiologists [38]. The incorporation of AI as a decision support tool has the potential to minimize oversight and improve the overall quality of fracture diagnosis.

Furthermore, AI algorithms can aid in detecting subtle or complex fractures that may be challenging to identify visually. Fractures with atypical presentations or overlapping structures can often be missed or misinterpreted by human observers. AI algorithms, particularly deep learning models, can effectively learn from diverse fracture patterns and identify even the most subtle fracture signs, and achieved high accuracy in detecting scaphoid fractures, which are notorious for being easily overlooked [39]. The integration of AI into fracture diagnosis enhances sensitivity and reduces the likelihood of missed fractures, ensuring that patients receive appropriate and timely treatment.

In addition to fracture detection, AI algorithms can assist in fracture classification, where errors in categorization can have significant implications for treatment planning. AI-based classification systems can standardize the interpretation of fracture characteristics, leading to consistent and objective results. This reduces the risk of misclassification and ensures appropriate treatment strategies [40]. By reducing classification errors, AI algorithms contribute to more accurate treatment decision-making and improved patient outcomes.

It is important to note that the integration of AI does not replace the role of radiologists or clinicians but rather complements their expertise. AI serves as a valuable tool to assist healthcare professionals in their decision-making processes, providing additional support and reducing the likelihood of errors and missed fractures.

However, despite the potential of AI in reducing diagnostic errors and missed fractures, challenges still exist. Developing accurate AI models requires large and diverse datasets, including both positive and negative fracture cases. The availability of such datasets for training AI algorithms can be a limitation in certain contexts. Furthermore, the generalizability of AI models across different patient populations, imaging devices, and clinical settings needs to be carefully evaluated to ensure consistent performance.

### Enhanced workflow and radiologist productivity

The integration of AI into fracture diagnosis has brought significant enhancements to workflow efficiency and radiologist productivity. By leveraging the capabilities of AI algorithms, time-consuming tasks can be automated, allowing radiologists to focus on more complex interpretations and providing opportunities for accelerated and streamlined diagnostic workflows.

One of the primary ways AI enhances workflow efficiency is through automated triage and prioritization of cases. AI algorithms can rapidly analyze imaging data and identify urgent or critical cases that require immediate attention. This feature allows radiologists to promptly address cases with suspected fractures, ensuring timely diagnosis and treatment. An AI system that successfully identified urgent findings in retinal fundus images, effectively triaging cases for further review by ophthalmologists [13]. The integration of similar AI-based triage systems in fracture diagnosis can significantly reduce the time spent on non-urgent cases and improve overall workflow efficiency.

AI algorithms can also automate repetitive tasks in the diagnostic process, such as image preprocessing, localization of fracture regions, and measurement of fracture parameters. These automated tasks save valuable time for radiologists, allowing them to focus on the interpretation and analysis of fracture images. For instance, an AI algorithm automatically detected and measured femoral fractures on radiographs, reducing the time required for these tasks. By alleviating radiologists from routine and time-consuming tasks, AI contributes to increased productivity and accelerated turnaround times [41].

In addition to task automation, AI algorithms can provide valuable decision support, assisting radiologists in the interpretation of fracture images. AI systems can generate computer-aided detection (CAD) markers or highlight regions of interest that may contain fractures, aiding radiologists in their assessments. This complementary support improves the efficiency and accuracy of fracture diagnosis. For example, this AI model achieved comparable performance to radiologists in detecting wrist fractures on X-rays [12]. The integration of AI-based CAD systems can expedite the interpretation process, reducing the burden on radiologists and enhancing productivity.

AI can also assist in automating repetitive and time-consuming tasks, such as fracture measurements and annotations. For example, deep learning algorithms have been developed to accurately measure fracture displacement and angulation, providing precise quantification without the need for manual measurements [42]. This automation not only saves time but also improves consistency and reduces inter-observer variability in fracture assessment.

Furthermore, AI algorithms can facilitate standardized reporting and documentation of fracture findings. By automatically extracting relevant information from imaging data and generating structured reports, AI systems help ensure consistency and completeness in reporting. This feature is particularly beneficial in busy clinical settings, where radiologists often face time constraints. For instance, this AI system generated structured breast imaging reports, reducing reporting time and improving report quality. Similar AI-based reporting systems can be implemented in fracture diagnosis, enabling efficient and standardized documentation of fracture findings [43].

In addition to these workflow enhancements, AI-powered triage systems can prioritize urgent or critical cases, ensuring that they receive prompt attention and reducing the risk of delays in diagnosis and treatment [44]. By flagging suspicious fractures or high-risk findings, AI algorithms can assist in timely patient management and facilitate the appropriate allocation of resources.

While AI enhances workflow efficiency and radiologist productivity, it is essential to maintain a collaborative approach between AI systems and human experts. Radiologists continue to play a critical role in the final interpretation and clinical decision-making process. AI acts as a valuable tool, providing support and accelerating workflows, but it does not replace the expertise and judgment of radiologists.

### Cost-effectiveness and resource optimization

The integration of AI into fracture diagnosis has the potential to improve cost-effectiveness and optimize healthcare resources. By streamlining the diagnostic process, reducing unnecessary procedures, and enhancing accuracy, AI can lead to significant cost savings and efficient resource allocation in orthopedic imaging departments.

#### Reducing unnecessary imaging studies

One of the key advantages of AI in fracture diagnosis is its potential to reduce the number of unnecessary imaging studies. AI algorithms can assist in identifying normal or non-fractured cases with high accuracy, enabling radiologists to focus on cases that require further evaluation. This targeted approach helps avoid unnecessary radiographs, reducing radiation exposure for patients and minimizing the associated costs. For example, this study developed an AI system that accurately classified normal and abnormal wrist radiographs, resulting in a significant reduction in the number of unnecessary wrist X-rays ordered in the emergency department [45]. By reducing the volume of unnecessary imaging studies, AI contributes to cost savings and optimized resource utilization.

#### Expediting diagnosis and reporting

Moreover, AI-based fracture detection systems can expedite the interpretation process, leading to shorter report turnaround times. Faster diagnosis and reporting enable prompt initiation of treatment and management decisions, potentially reducing hospital stays and associated costs using an AI system for detecting hip fractures on radiographs [46].

#### Optimizing resource allocation

AI algorithms can also aid in optimizing resource allocation by facilitating workload distribution and improving radiologist efficiency. By automating routine tasks, such as image preprocessing and fracture localization, AI algorithms free up valuable radiologist time, enabling them to focus on more complex cases and critical decision-making. This improved workflow efficiency ensures optimal utilization of radiologists’ expertise and minimizes the need for additional staffing. For example, this study demonstrated that the integration of an AI system for detecting wrist fractures improved radiologist efficiency by reducing the average time spent on each case [47]. This increased efficiency translates into optimized resource allocation and improved cost-effectiveness within the healthcare system.

#### Improving patient outcomes

In addition to direct cost savings, AI-based fracture diagnosis can contribute to indirect cost reductions by improving patient outcomes and reducing the need for subsequent interventions. Accurate and timely fracture diagnosis enables appropriate treatment planning and facilitates early intervention, which can prevent complications and reduce the need for extensive surgical procedures or hospital readmissions. For instance, a study showed that the implementation of an AI system for diagnosing distal radius fractures led to a reduction in surgical treatment rates due to improved conservative management decisions [48]. By optimizing treatment strategies and minimizing the need for additional interventions, AI-based fracture diagnosis can contribute to overall cost-effectiveness in orthopedic care.

## Challenges and future directions

### Ethical considerations and legal implications of AI adoption

The integration of AI into fracture diagnosis raises important ethical considerations and legal implications that need to be carefully addressed. As AI algorithms become more prevalent in healthcare settings, it is crucial to ensure that their adoption adheres to ethical principles, respects patient rights, maintains privacy and confidentiality, and complies with relevant legal frameworks.

### Transparency and explainability

One of the primary ethical considerations in AI adoption is the transparency and explainability of AI algorithms. It is essential for AI systems used in fracture diagnosis to provide transparent and interpretable outputs, enabling radiologists and healthcare professionals to understand how the algorithms arrive at their conclusions. This transparency promotes trust, accountability, and clinical acceptance. Researchers and developers must strive to enhance the explainability of AI algorithms, employing techniques such as model interpretability and visualizations to elucidate the decision-making process. Ethical guidelines, such as those proposed by the European Commission’s High-Level Expert Group on AI emphasize the importance of transparency and explainability in AI systems [49].

### Patient privacy and data protection

Patient privacy and data protection are critical aspects of AI adoption. AI algorithms in fracture diagnosis often require access to large volumes of patient data, including medical images and clinical information. It is essential to ensure that patient data is handled securely, following applicable privacy regulations and institutional policies. Anonymization and de-identification techniques can be employed to protect patient privacy, and strict data governance protocols should be in place to safeguard against data breaches. Compliance with data protection laws, such as the Health Insurance Portability and Accountability Act (HIPAA) in the United States, is essential to ensure ethical AI implementation [50].

### Bias and fairness

Bias and fairness in AI algorithms are ethical considerations that demand careful attention. AI algorithms trained on biased or unrepresentative datasets may perpetuate existing disparities and inequalities in healthcare. To mitigate this, it is crucial to ensure diverse and inclusive training datasets that adequately represent different population groups. Regular monitoring and evaluation of AI systems for potential bias and fairness issues should be conducted, and steps should be taken to rectify any identified biases. Ethical frameworks, such as the ACM Code of Ethics and Professional Conduct, highlight the importance of fairness, accountability, and social responsibility in the development and deployment of AI technologies [51].

### Legal implications

Legal implications associated with AI adoption in fracture diagnosis include liability, malpractice, and regulatory compliance. Determining the responsibility and liability in cases where AI systems are involved can be complex. Clear guidelines and regulations are necessary to define the roles and responsibilities of AI developers, healthcare providers, and radiologists. Additionally, legal frameworks need to address issues related to medical malpractice and potential errors or adverse outcomes resulting from AI-assisted diagnoses. Regulations, such as the Medical Device Regulation (MDR) in the European Union, may provide guidance on the certification, safety, and accountability of AI systems in healthcare.

### Ethical review boards and institutional ethics committees

Ethical review boards and institutional ethics committees play a crucial role in ensuring the ethical and legal compliance of AI adoption in fracture diagnosis. These bodies should oversee the implementation of AI systems, assess their potential risks and benefits, and ensure that appropriate consent procedures and patient information protocols are in place. Collaboration among healthcare professionals, researchers, policymakers, and legal experts is essential to develop comprehensive ethical guidelines and legal frameworks that address the unique considerations associated with AI adoption.

### Federated learning

In the context of AI, federated learning is emerging as a promising approach to address both data privacy and security concerns. Federated learning allows AI models to be trained across multiple decentralized institutions while keeping the patient data locally stored and protected. By keeping the data at its source, federated learning minimizes the need for data sharing, thus reducing privacy risks. This approach ensures that sensitive patient information remains under the control of the data owner while enabling collaborative model training and knowledge sharing.

### Governance frameworks

Furthermore, robust governance frameworks are necessary to ensure responsible data management and address privacy and security concerns effectively. Healthcare institutions should establish clear policies and guidelines for data handling, including data access, sharing, and retention. Ethical review boards and data governance committees can provide oversight and ensure compliance with privacy and security regulations. Regular staff training on data privacy and security protocols is essential to foster a culture of awareness and responsibility among healthcare professionals involved in AI-based fracture diagnosis.

### Public transparency and communication

Public transparency and communication are crucial in addressing data privacy and security concerns. Healthcare organizations should clearly communicate their data privacy policies, data usage practices, and security measures to patients, ensuring transparency and informed consent. Open dialogue with patients and the public regarding the benefits, risks, and safeguards associated with AI technologies builds trust and encourages patient engagement in their healthcare journey.

In summary, the integration of AI into fracture diagnosis necessitates robust data privacy and security measures. Adherence to privacy regulations, data anonymization techniques, strong data security protocols, and the exploration of privacy-preserving approaches like federated learning are essential to protect patient confidentiality. A comprehensive governance framework and transparent communication practices ensure responsible and accountable data management. By addressing data privacy and security concerns effectively, AI technologies can be deployed in fracture diagnosis while upholding patient privacy rights and maintaining public trust.

## Conclusion

AI has emerged as a transformative technology in fracture diagnosis, offering the potential to enhance accuracy, efficiency, and clinical decision-making. The integration of deep learning models, CNNs and RNNs, NLP techniques, and multi-modal imaging approaches has shown promising results. While challenges remain, addressing biases, ensuring data privacy and security, and promoting transparency are crucial for the responsible and equitable adoption of AI in fracture diagnosis.

Some of the key findings from the review:

- AI models, particularly deep learning algorithms, have shown remarkable performance in fracture detection and classification, rivaling, or surpassing human experts in accuracy and efficiency.
- CNNs have emerged as a powerful approach for fracture detection, demonstrating high sensitivity and specificity. Their ability to learn complex patterns in X-ray images has facilitated automated fracture detection and localization.
- The incorporation of RNNs has shown promise in fracture classification tasks, leveraging sequential information and capturing temporal dependencies in X-ray series.
- NLP techniques have been utilized to extract clinical information from radiology reports, facilitating automated fracture diagnosis and improving the efficiency of radiologists.
- Integration of AI with other imaging modalities, such as CT and MRI, has the potential to enhance fracture diagnosis accuracy and provide a comprehensive assessment of complex fractures.
- The adoption of AI in fracture diagnosis has demonstrated significant improvements in accuracy, leading to reduced diagnostic errors and missed fractures. AI-based systems can serve as valuable decision-support tools for radiologists and orthopedic surgeons.
- The implementation of AI technologies has shown the potential to enhance workflow and radiologist productivity by automating time-consuming tasks, such as image annotation and report generation.
- Cost-effectiveness and resource optimization are other potential benefits of AI adoption in fracture diagnosis. AI-based systems can help reduce unnecessary imaging tests, decrease patient wait times, and optimize resource allocation.
- Ethical considerations and legal implications of AI adoption in fracture diagnosis must be carefully addressed. Issues such as bias, fairness, privacy, and accountability require attention to ensure the responsible deployment of AI technologies.
- Future research directions in this field include multi-modal fusion, real-time fracture detection, automated 3D fracture reconstruction, clinical decision support systems, longitudinal fracture analysis, clinical workflow optimization, and data sharing.
